# The Book World of Medicine and Science

**Published:** 1893-06-24

**Authors:** 


### The Book World of Medicine and Science.

### BOOK REVIEWS.

Sir Morell Mackenzie, Physician and Operator: A Memoir, Compiled and Edited from Private Papers and Personal Reminiscences, by Rev. H. R. Haweis, M.A., author of "Music and Morals," "My Musical Life, &c. (London: W. H. Allen and Co. (Limited), 13, Waterloo Place.)

One must regard Sir Morell Mackenzie as having forestalled Mr. Haweis in the most inconsiderate manner by the publication of his "Frederick the Noble." It is all but certain that there would have been no memoir of the "Physician and Operator" beyond the ordinary obituary notices of the professional and daily press if it had not been for his attendance at that death bed on which the eyes of all Europe were fixed. The interest of this volume lies in what additional light is thrown on the Emperor Frederick's illness, the rest is mere padding, and rather inferior padding at that. Does the world really care to know Morell Mackenzie's tastes in food, his favourite games, to be informed that he was generous to the point of extravagance in gratifying the wishes of his wife and daughters? It may be a satisfaction to fond parents of female] infants to be told that Sir Morell Mackenzie's daughters were named Ethel, Olga, and Hilda; but when that interesting piece of information was given once, why need it be repeated on the next page. Mackenzie's life was that of a successful specialist, in no way different from a score of others. He had to fight for recognition, he won it, he made a large income, he attended the poor, and many who were not poor, without fee or reward; he established a hospital for the practice of his specialty; and all is said except the fact that he, an Englishman, was sent to share with German surgeons, the case of a German prince attacked by a fatal disease.

Was the disease fatal in itself, or was it made so by maltreatment? That question was asked when the Emperor died, and the pages of "Frederick the Noble" showed more readiness and acrimony than were at all becoming in bringing grave insinuations against the German specialists. Mackenzie's repute in the professional world was not increased by the evidence his own book gave of irritability and personal feeling at a crisis when propriety demanded an absorbed interest in his patient's condition; but he at least suppressed the distinct accusation against Professor Von Bergmann of having largely added to the Emperor's agony, and hastened, if he did not cause, his death, which is given in the present work. Why has this been done? It will do nothing to alter the opinion in which either the Briton or the Teuton is held, for all who interested themselves in the question made up their mind on it years ago; while it may, it must, cause the most bitter grief to the royal lady to whom Sir Morell Mackenzie owed his anxious but honourable charge, and whose feelings, we fully believe, he would have most wished to spare. In short, this is a book the publication of which is most regrettable. For the most part it is trivial, and the rest is ungenerous and in some respects

THE BOOK WORLD OF MEDICINE AND SCIENCE.—*(continued).*

cruel. Mr. Haweis has written to the newspapers saying that he and the publishers alike were willing to withdraw the book from circulation, but Sir Morell Mackenzie's family would not consent. This is all very well, but to accuse the Mackenzie family of a lack of taste and judgment does not exonerate Mr. Haweis. If they consented, did not he actually compile, and guarantee by his hitherto honourable name, a book whose inanity is tempered only by indiscretion if not by malice?

An Introduction to Practical Bacteriology. By Dr. W. Migula. Translated by M. Campbell, and Edited by H. J. Campbell, M.D., M.R.C.P. (Swan Sonnenschein and Co.)

They come, and yet they come apace, these new works on bacteriology; whether the demand is equal to the supply is a question which concerns the compiler and the publisher rather than the purchaser. And if the former are satisfied with the results, there is no reason why the latter should quarrel with the hand that panders to his appetite. Moreover many a student of bacteriology is, alas, too often no student of German, and hence unable to read bacteriological works in what we may call the mother language of the science, for in Germany this science was given birth, and in German its annals have been written. This little volume affords no exception to the rule, since its author was Dr. Migula, of Karlsruhe. Neither he, nor the editor of the English version, namely, Dr. Campbell, of Guy's Hospital, can be considered professional bacteriologists; both of them, however, are teachers of some experience, and their volume is well adapted to meet the requirements of students commencing the study of bacteria. As a book to be actually used in the laboratory, it does not appeal to us as meeting all the requirements either of the teacher or of the taught; if in part it had been drawn up on the lines of Foster and Langley's practical physiology or of Schäfer's histology, the student might have derived greater profit to himself, with less assistance from the demonstrator. It appears to us that this work of Migula's is more what may be called a book for the fire-side student than for the practical one in a working laboratory, and, strange to say, it was peculiarly to this latter class that the author professedly dedicated his work. We must admit, however, that though failing in his avowed ambition, he has unwittingly produced a text book of no small merit, on a subject which does not readily lend itself to compression within the narrow limits of 238 pages. Bacillus anthracis, and bacillus tuberculosis, the bacteriological equivalents of mensa and filius of the Latin grammar, are honoured by separate chapters; the bacteriological examination of water in regard to its infective properties, as its importance merits, holds a deservedly conspicuous position in this book. The infective properties of contaminated articles of diet, such as meat and milk, subjects at least as important to the physician, are almost untouched on by the author; and although the subject of sterilisation in its general principles is ably handed, nevertheless its practical and special adaptation to surgical methods is left untouched. Faults of omission, however, are easy of demonstration where an author's object has

THE BOOK WORLD OF MEDICINE AND SCIENCE—(*continued*).

been brevity, faults of commission are few and far between; we must, therefore congratulate the author the translator and ourselves on the valuable little addition to our bacteriological library.

### JUNE REVIEWS.

THREE contributions in the NEW REVIEW on "Crime and Punishment" by Sir Henry Hawkins, Mr. Hopwood, Q.C., and Mr. Poland, Q.C., are well worth studying. Dr. Richardson presents a report of the doings of the society, of which he is president, for the improvement of public slaughter-houses. The subject is not a very attractive one, but it is undoubtedly of great moment in the preservation of public health. It is not very cheering to learn that "the Jewish inspectors reject as improper food for their people 35 per cent. of oxen, 25 per cent. of calves, and 25 per cent. of sheep. The whole of this rejected sustenance finds its way into the Gentile organism." Under the new and model abattoir system slaughtering will be conducted solely by well-paid skilled experts belonging to the abattoirs, and will be almost, if not altogther, painless. The preliminary inspection of the animals, and the scrupulous cleanliness of the lair and general premises of the abattoir are points on which much stress is laid, and which appear to be generally neglected.

THE CONTEMPORARY REVIEW has not much that is striking this month, but is redeemed by a fine article on "The Prospects of the Civilised World," by the Rev. J. Llewellyn Davies, whose protest against Mr. Pearson's cynical pessimism is both wholesome and refreshing.

### CANCER FOCI.

THE *Birmingham Medical Review* for December, 1892, contains a very interesting paper by Mr. Law Webb on the etiology of cancer, in which he adduces evidence to show that cancer sometimes occurs in successive inmates of a house, or is particularly frequent in a special group of houses. The facts stated have all come under his own personal observation, and they are by no means isolated. General practitioners can greatly help in elucidating this point.

